# Honeycomb-like Structured Film, a Novel Therapeutic Device, Suppresses Tumor Growth in an In Vivo Ovarian Cancer Model

**DOI:** 10.3390/cancers15010237

**Published:** 2022-12-30

**Authors:** Tsuyoshi Ohta, Masaru Tanaka, Seitaro Taki, Hiroyuki Nakagawa, Satoru Nagase

**Affiliations:** 1 Department of Obstetrics and Gynecology, Faculty of Medicine, Yamagata University, Yamagata 990-9585, Japan; 2 Institute for Materials Chemistry and Engineering, Kyushu University, CE41 744 Motooka, Nishi, Fukuoka 819-0395, Japan; 3 Toyoda Gosei Co., Ltd., 1-1, Higashitakasuka, Futatudera, Miwa-cho, Ama-gun, Aichi 490-1207, Japan

**Keywords:** therapeutic device, ovarian cancer, tumor growth

## Abstract

**Simple Summary:**

Ovarian cancer cell dissemination can lead to inoperability and death in patients with advanced ovarian cancer. Honeycomb-like structured films (HCFs) are three-dimensional (3D) porous scaffolds fabricated from biodegradable polymers and have been widely used in tissue engineering. We assessed whether HCFs could be a novel strategy to inhibit residual tumor growth after surgery. Here, we show that HCFs can remarkably suppress tumor growth in an in vivo ovarian cancer model. RNA sequencing of paired tumors treated with HCFs and control tumors that were treated without films demonstrated that HCFs induced abnormal focal adhesion and cell morphological change, subsequently inhibiting differentiation, proliferation and motility in ovarian cancer cells. Our data suggest that HCFs could inhibit residual tumor growth after surgery, reduce surgical invasiveness, and improve prognosis for patients with advanced ovarian cancer.

**Abstract:**

Ovarian cancer cell dissemination can lead to the mortality of patients with advanced ovarian cancer. Complete surgery for no gross residual disease contributes to a more favorable prognosis than that of patients with residual disease. HCFs have highly regular porous structures and their 3D porous structures act as scaffolds for cell adhesion. HCFs are fabricated from biodegradable polymers and have been widely used in tissue engineering. This study aimed to show that HCFs suppress tumor growth in an in vivo ovarian cancer model. The HCF pore sizes had a significant influence on tumor growth inhibition, and HCFs induced morphological changes that rounded out ovarian cancer cells. Furthermore, we identified gene ontology (GO) terms and clusters of genes downregulated by HCFs. qPCR analysis demonstrated that a honeycomb structure downregulated the expression of CXCL2, FOXC1, MMP14, and SNAI2, which are involved in cell proliferation, migration, invasion, angiogenesis, focal adhesion, extracellular matrix (ECM), and epithelial–mesenchymal transition (EMT). Collectively, HCFs induced abnormal focal adhesion and cell morphological changes, subsequently inhibiting the differentiation, proliferation and motility of ovarian cancer cells. Our data suggest that HCFs could be a novel device for inhibiting residual tumor growth after surgery, and could reduce surgical invasiveness and improve the prognosis for patients with advanced ovarian cancer.

## 1. Introduction

Ovarian cancer is the most lethal gynecological cancer, and approximately 70% of patients are diagnosed at an advanced stage [1]. The standard therapy for ovarian cancer is debulking surgery, followed by platinum-based chemotherapy [2]. The survival rate of patients with complete surgery to no gross residual disease is significantly higher than that of patients with optimal surgery (residual disease < 1 cm) and suboptimal surgery (residual disease > 1 cm) [3,4]. Widespread peritoneal dissemination is an advanced ovarian cancer hallmark; extensive surgery including bowel resection, diaphragmatic peritonectomy, and splenectomy is necessary to achieve complete surgery [5,6,7,8]. The rate of complete surgery for advanced ovarian cancer is approximately 60%, even in oncological referral centers [9]. Although extensive surgery with no residual disease results in better survival than comprehensive surgery with residual disease in patients with ovarian cancer, it is associated with a higher incidence of postoperative complications [10] and reduced quality of life [11]. Therefore, we believe that a new device is required to control residual tumors after primary debulking surgery (PDS). Moreover, this new device should be able to reduce surgical invasiveness and improve the prognosis of patients with advanced ovarian cancer. 

HCFs are 3D porous scaffolds fabricated from biodegradable polymers, and have been widely used as a temporary ECM; these play critical roles in tissue engineering [12]. Most tissue-derived cells are anchorage-dependent and require attachment to solid surfaces for viability and growth. Therefore, cell adhesion to a surface is the initial event, and is critical for subsequent events such as cell migration, spread, and differentiation. HCFs with highly regular porous structures can be prepared under humid casting conditions [13]. Pore sizes on the surface of scaffolds have a significant influence on the morphology, proliferation, differentiation, and function of various normal cells [14,15,16]. HCFs with a pore size of 5 μm induced morphological changes to spheroids in hepatocytes, and increased liver-specific functions such as albumin secretion and urea synthesis [17]. HCFs with subcellular-sized pores promoted neural stem/progenitor cell proliferation, while preventing their differentiation into neurons [18]. The endothelial cells on the HCFs, with a pore size of 5 μm, exhibited greater spreading and flatting than those on the flat film and the HCFs induced cell proliferation and extracellular matrix (ECMs) production [15]. In normal cells, HCFs enhance cell viability, growth, function, and differentiation, whereas human cancer cells cultured on HCFs exhibit reduced cell growth and motility [19]. Cancer cell growth on HCFs was lower than that of cells on control flat films, and over 50% inhibition was observed in 27 of 58 cancer cell lines, including gall bladder, lung, oral, stomach, colon, pancreatic and ovarian cancers [19]. However, the mechanism underlying cancer cell growth inhibition by HCFs remains unclear. Moreover, there are no reports examining the effect of HCFs on tumor growth in vivo.

Therefore, in this study, we examined the effects of HCFs on tumor growth in ovarian cancer using a mouse model. We also examined the mechanisms underlying tumor growth inhibition by HCFs using RNA sequencing. Moreover, we assessed the possibility using HCFs as post-surgical material in the patients with residual disease.

## 2. Materials and Methods

### 2.1. Cell Cultures

The SKOV3ip1 cell line was generated from ascites developed in nu/nu mice via intraperitoneal injection of the human ovarian carcinoma cell line SKOV3 [20]. SKOV3ip1 cells were provided by Dr. Hung at the MD Anderson Cancer Center (Houston, TX, USA). ES-2 was provided by Prof. Yaegashi of the Department of Gynecology and Obstetrics, Tohoku University Graduate School of Medicine (Sendai, Japan). The SKOV3ip1 cell line was cultured at 37 °C in medium composed of 1:1 combination of MCDB105 (Sigma-Aldrich; Merck KGaA, Tokyo, Japan) and Medium199 (Sigma-Aldrich; Merck KGaA) with 10% fetal bovine serum (FBS) (Sigma-Aldrich; Merck KGaA) and 1% penicillin-streptomycin (Sigma-Aldrich; Merck KGaA) in a humidified atmosphere of 95% air and 5% CO_2_. ES-2 cells were maintained in DMEM/F12 medium (Thermo Fisher Scientific, Inc., Tokyo, Japan), supplemented with 10% FBS and 1% penicillin–streptomycin at 37 °C in a humidified atmosphere with 5% CO_2_.

### 2.2. Preparation of HCFs

Porous polyurethane HCFs were produced by foaming urethane-coated films in a steam atmosphere. The pore size was regulated by changing parameters such as temperature and humidity. HCFs were prepared as films with a pore size of 5–8 μm (small), 8–12 μm (medium), and 12–16 μm (large). Flat films without pores were prepared by casting the polymer solution under dry conditions. The HCFs and flat films were reproducible and provided by Toyoda Gosei Co., Ltd., (Aichi, Japan).

### 2.3. Subcutaneous Xenograft Model and Treatment of HCFs in vivo

All the procedures involving animals were approved by the animal care committee of Yamagata University (approval no. 31073) and conducted per the institutional and Japanese government guidelines for animal experiments. SKOV3ip1 and ES-2 cells were harvested in 0.25% trypsin/phosphate-buffered saline (PBS)/EDTA, washed once with medium and PBS, and resuspended in PBS at 1 × 10^6^ cells/100 µL. SKOV3ip1 or ES-2 cells (2 × 10^6^) were injected subcutaneously into the flanks of 6-week-old female BALB/cAJcl-nu/nu mice. In the subcutaneous model, once the tumor length reached 10 mm after implantation, a subcutaneous incision was made in the mice under anesthesia, polyurethane flat films without pores or polyurethane HCFs with small (5–8 μm), medium (8–12 μm), or large (12–16 μm) pores were applied under the formed tumor’s surface, and then the skin was sutured. Mice that underwent surgical manipulation without film application served as controls (sham). We did not assess the tumor weight when tumor reached 10 mm because the complete removal of tumor would disrupt the nutrient blood vessels formed from tumor to skin of mice. Our preliminary data demonstrated that the days of significant tumor size causing distress mice in sham groups of SKOV3ip1 and ES2 were 24 days and 21 days after surgery, respectively. Therefore, the tumors formed form SKOV3ip1 were removed and weighed 24 days after surgery in mice, while the tumors formed from ES-2 were removed and weighted 21 days after surgery in mice. The difference in the number of days before removing the tumors is due to the different rate of tumor formation between SKOVip1 and ES-2 cells.

### 2.4. RNA Sequencing and RNA-Sequencing Data Analysis

Tissue samples were collected in sterile tubes and stored at –80 °C. Tumors were homogenized in RLT buffer and total RNA was isolated using the RNeasy Mini Kit (QIAGEN, Hilden, Germany). Total RNA was used for library preparation, achieved using rRNA-depleted RNA and a NEBNext Ultra Directional RNA Library Prep Kit for Illumina (NEB), according to the manufacturer’s recommendations. The samples were sequenced on an Illumina HiSeq X TEN platform (2 × 150-bp paired-end reads) in Novogene, China. Details of the procedure are described in a previous study [21]. The reference genome and gene model annotation files were downloaded from the genome website (NCBI/UCSC/Ensembl). The reference genome index was built using Hisat2 v2.0.5, and clean paired-end reads were aligned to the reference genome using Hisat2 v2.0.5. The read counts were adjusted using the edgeR program package through one scaling normalized factor for each sequenced library before differential gene expression analysis,. Differential expression analysis under the two conditions was performed using the edgeR R package (version 3.16.5). P values were adjusted using the Benjamini and Hochberg method. A corrected P-value of 0.05 and an absolute fold change of 1 were set as the threshold for significant differential expression. To identify the correlation between differences, we clustered different samples using expression level FPKM and visualized the correlation via the hierarchical clustering distance method and the functions heatmap, self-organization mapping (SOM) and k-means using silhouette coefficient to adapt the optimal classification with default parameter in R. A volcano diagram can visually show the whole distribution of differential expression genes (DEGs). For the samples with biological replicates, the threshold of differential expression genes was padj < 0.05. For the samples without biological replicates, the threshold of differential expression genes was |log2 (fold change)| > 1 and *p*-value < 0.005. DEGs between HCFs-treated and control were investigated using Gene Set Enrichment Analysis (GSEA) to identify enriched biological pathways, as defined by the set of genes between the two treatments. GSEA can detect subtle changes in the expression levels. We used the local version of the GSEA analysis tool (http://www.broadinstitute.org/gsea/index.jsp, accessed on 4 March 2020). Gene ontology (GO) terms were performed using the clusterProfiler R package.

### 2.5. Real-Time PCR Analysis

All primers and probes used in the quantitative real-time PCR (qRT-PCR) analysis were obtained from Takara Bio Inc. (Shiga, Japan), and are described in Supplementary Table S1. qRT-PCR was performed using ABI Prism 7300 (Applied Biosystems, Life Technologies, Carlsbad, CA, USA), according to the manufacturer’s protocol. All PCR procedures were performed in triplicate. Reactions involved an initial incubation for 2 min at 50 °C, then 10 min at 95 °C, followed by 50 cycles of 95 °C for 15 s, and 60 °C for 1 min. The 18S gene was used for the normalization of the cDNA templates. Quantification was performed using the standard curve method. A standard curve was generated from a dilution series constructed from the cDNA extracted from SKOV3ip1.

### 2.6. Scanning Electron Microscopy (SEM) Observation

SKOV3ip1 cells were cultured on flat, small, medium, or large HFSs for 1 week. The samples were dehydrated by washing in increasing concentrations of ethanol and subsequently dried using a critical point dryer (HCP-2, Hitachi, Tokyo, Japan). The dried samples were mounted on aluminium stages with a double-sided adhesive tape and coated with a 5 nm layer of palladium goldusing an ion sputter coater (E-1030, Hitachi, Tokyo, Japan). All samples were observed using SEM (Hitachi S-3500N, Tokyo, Japan).

### 2.7. Confocal Laser-Scanning Microscopy (CLSM) Observation

To assess the SKOV3ip1 and ES2 cell size, cells were cultured on dishes for 1 day. After culture, the cells were fixed in 4% paraformaldehyde in PBS (FUJIFILM Wako Pure ChemicalCorporation, Osaka, Japan) for 10 min at 37 °C and treated three times with 1% Triton X-100 (MP Biomedicals, LLC) in PBS for 10 min at room temperature. After permeabilization, the cells were stained with mouse anti-vinculin monoclonal antibody (Millipore, Billerica, MA, USA) for 2 h at 37 °C, followed by treatment with Alexa Fluor 546-conjugated anti-mouse IgG antibody (Invitrogen, Carlsbad, CA, USA) and Alexa Fluor 488-conjugated phalloidin (Invitrogen) for 1 h at 37 °C in the dark. Prolonged gold antifade reagent with DAPI (Invitrogen) was used to mount the slides and to counterstain the cell nuclei, respectively. The observations were made using a CLSM (Olympus, Tokyo, Japan).

### 2.8. Statistical Analysis

Statistical analysis was performed using GraphPad Prism software (version 8.0; GraphPad Software, Inc., San Diego, CA, USA). Data are presented as mean ± SE. Comparisons between two groups were performed via an unpaired Student’s *t*-test. One-way followed by the Bonferroni post hoc test was used to compare differences between multiple groups. *p* < 0.05 was considered a statistically significant difference and is represented by an asterisk in the figures.

## 3. Results

### 3.1. Effects of HCFs on Tumor Growth in Ovarian Cancer

**Figure 1:**
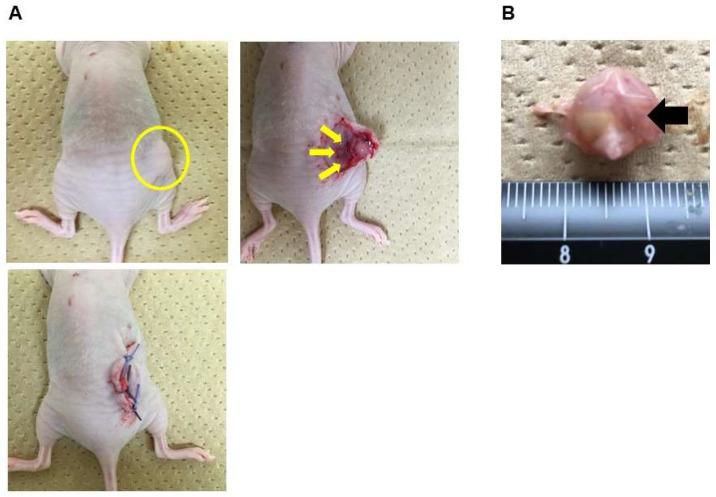
Representative surgical procedures. (**A**) Confirmation that the tumor’s length reached 10 mm after implantation (yellow circle), a subcutaneous incision was made in the mice under anesthesia, and polyurethane flat films without pores or polyurethane HCFs with small (5–8 μm), medium (8–12 μm), or large (12–16 μm) pores were applied under the surface of the formed tumor (yellow arrow), and the skin was sutured. (**B**) During tumor removal, the HCFs (black arrow) were affixed to the tumor surface.

**Figure 2:**
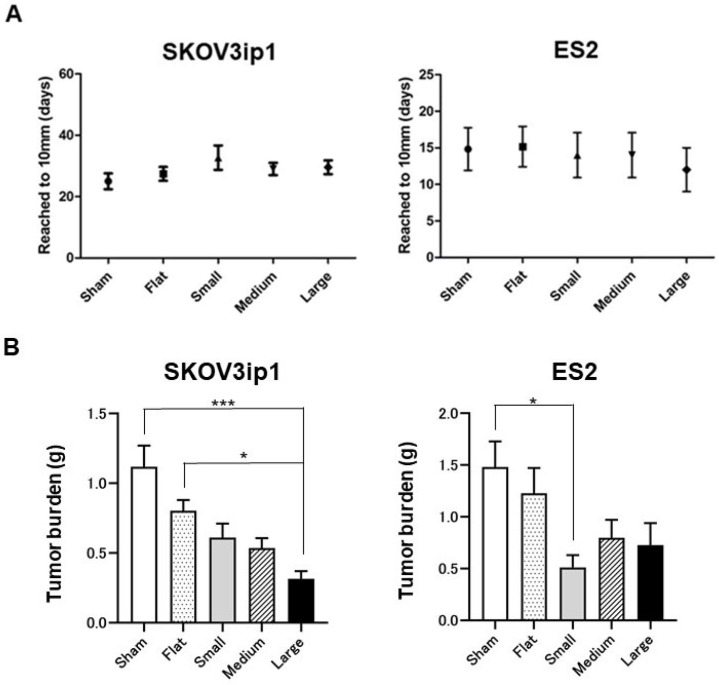
Effects of HCFs on tumor growth in ovarian cancer. (**A**) Number of days to achieve a tumor size of 10 mm. The number of days to achieve a tumor size of 10 mm was the control tumor (sham; ●) for 25.0 ± 5.2, flat films (flat; ■) for 27.4 ± 5.2, HCFs with small pores (small; ▲) for 32.7 ± 9.7, HCFs with medium pores (medium; ▼) for 29.0 ± 4.5, and HCFs with large pores (large; ◆) for 29.6 ± 5.5 in SKOV3ip1, and sham (●) for 14.8 ± 6.5, flat (■) for 16.0 ± 7.2, small (▲) for 14.0 ± 6.1, medium (▼) for 14.0 ± 6.1, and large (◆) for 12.0 ± 5.2 in ES2, Values of the days are shown as the mean ± SE. (**B**) The tumors formed form SKOV3ip1 were removed and weighed 24 days after surgery in mice, while the tumors formed from ES-2 were removed and weighted 21 days after surgery in mice. Values of tumor weights are shown as the mean ± SE. Significant differences are indicated by asterisks. ***, *p* < 0.001, *, *p* < 0.05.

We tested whether HCFs could suppress tumor growth in an ovarian cancer mouse model. The representative surgical procedures are shown in Figure 1. The number of days needed to achieve a tumor size of 10 mm was the control tumor (sham) for 25.0 ± 5.2, flat films (flat) for 27.4 ± 5.2, HCFs with small pores (small) for 32.7 ± 9.7, HCFs with medium pores (medium) for 29.0 ± 4.5, and HCFs with large pores (large) for 29.6 ± 5.5 in SKOV3ip1, and sham for 14.8 ± 6.5, flat for 16.0 ± 7.2, small for 14.0 ± 6.1, medium for 14.0 ± 6.1, and large for 12.0 ± 5.2 in ES2, which were not significantly different in each group (Figure 2A). HCFs with large pores resulted in a significant decrease in tumor weight compared to those in the control tumor (sham) or tumors treated with the flat films (flat) in SKOV3ip1 cells (Figure 2B, left panel). The tumor weight on HCFs with small pores was significantly lower than that on the control tumor (sham) in ES2 cells (Figure 2B, right panel). These results suggest that the pore sizes of HCFs has a significant influence on tumor growth suppression in an ovarian cancer mouse model.

### 3.2. Morphological Changes in Ovarian Cancer Cells Cultured on HCFs

**Figure 3:**
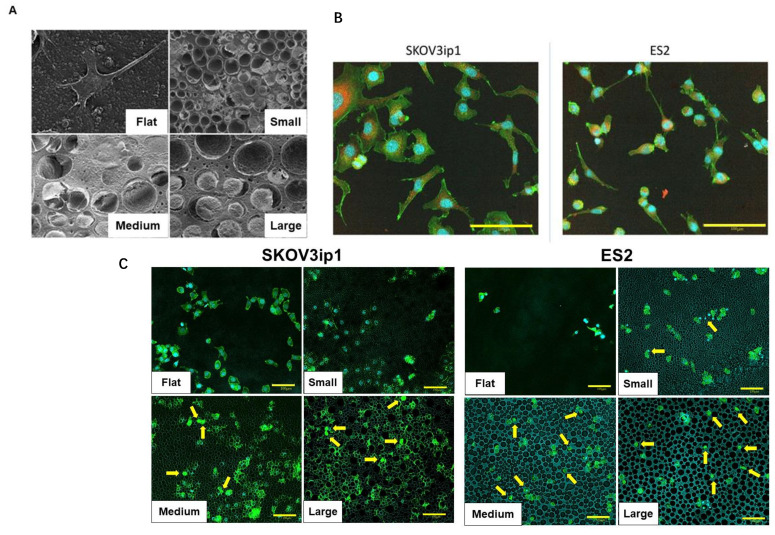
SEM and CLSM images of ovarian cancer cell lines. (**A**) SEM images of SKOV3ip1 cells morphologies on the flat films (Flat), films with a pore size of 5–8μm (Small), 8–12 μm (Medium), and 12–16 μm (Large). (**B**) CLSM images of SKOV3ip1 and ES2 cells showed the merged staining for the nucleus (blue), actin (green), and vinculin (red). All scale bars (yellow line) are 100 μm. (**C**) The numbers of cells settled into the pores in SKOV3ip1 and ES2 cells were assessed by CLSM images. The yellow arrow indicates those cells.

We examined whether the pore size of HCFs affected the morphology of ovarian cancer cells using SEM. On flat films, SKOV3ip1 cells exhibited a typical monolayer morphology (Figure 3A, Flat). The cells cultured on HCFs with large pores appeared to be rounded and settled into the pores (Figure 3A, Large). We assessed the size of the SKOV3ip1 and ES2 cells using CLSM. The average cellular area of SKOV3ip1 and ES2 cells were approximately 1000–2000 μm^2^ and 300–500 μm^2^, respectively (Figure 3B). We also examined if there were differences in the number of cells settled into the pores in flat membrane and each pore size of HCFs by CLSM. The numbers of cells that infiltrated the small pores of HCFs were low in SKOV3ip1, whereas a few cells were observed in ES2 cells (Figure 3C). The numbers of those cells increased in HCFs with medium and large pores in the two cell lines. These findings indicated that HCFs suppressed tumor growth by inducing morphological changes; however, the inhibitory effects in HCFs of different pore sizes could not be explained by the size of cancer cells.

### 3.3. The Mechanism of Tumorigenic Inhibitory Effect by HCFs

**Figure 4:**
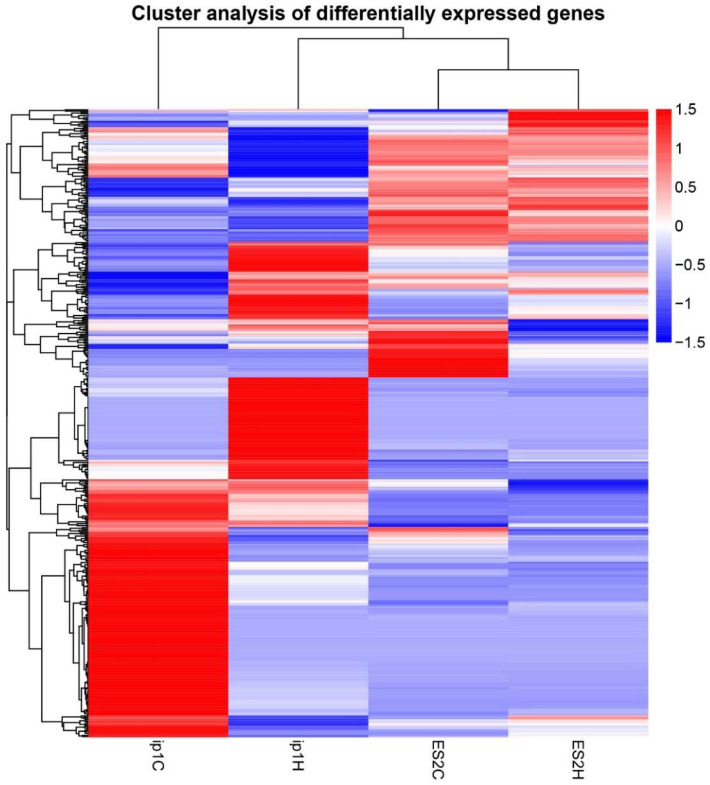
Cluster analysis of differential expression genes in paired tumors treated with HCFs and the control tumor not treated with films. Tumor tissues were obtained from the control (ip1C) and HCFs with 12–16 μm pores (ip1H) in SKOV3ip1 cells, and from the control (ES2C) and HCFs with 5–8 μm pores (ES2H) in ES2 cells. The hierarchical clustering analysis was performed with the log10(FPKM+1) of union differential expression genes of all comparison groups under different experimental conditions. Data colored in the red–white–blue scheme indicate a relatively higher, average, and lower expression, respectively.

**Figure 5:**
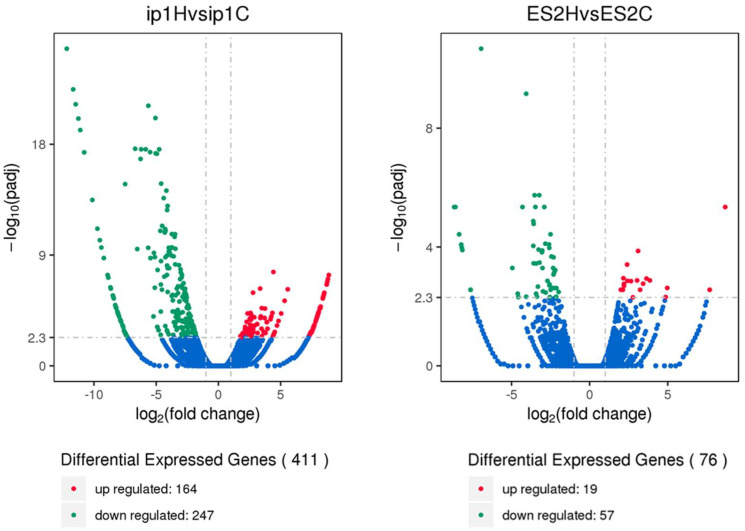
Volcano diagram of differential expression genes in paired tumors treated with HCFs and the control tumor not treated with films. Tumor tissues were obtained from the control (ip1C) and HCFs with 1216 μm pores (ip1H) in SKOV3ip1 cells, and from the control (ES2C) and HCFs with 58 μm pores (ES2H) in ES2 cells. The horizontal axis represents the fold change of genes in different samples. The vertical axis represents the statistically significant degree of changes in gene expression levels, the more significant the difference, and the smaller the corrected *p*-value, the bigger −log10 (corrected *p*-value). The point represents a gene, blue dots indicate no significant difference in genes, red dots indicate upregulated differentially expressed genes, and green dots indicate downregulated differentially expressed genes.

To identify the mechanisms underlying tumor growth inhibition by HCFs, we performed RNA sequencing of paired tumors treated with HCFs and the control tumors that were not treated with films. We used tumor tissues obtained from the control (ip1C) and HCFs with large pores (ip1H) in SKOV3ip1 cells, and from the control (ES2C) and HCFs with small pores (ES2H) in ES2 cells. Hierarchical clustering analysis revealed significant differences in the levels of gene expression between the control (ip1C) and tumors treated with HCFs (ip1H) in SKOV3ip1 cells (Figure 4, left two lanes). However, the gene expression patterns between the control (ES2C) and tumors treated with HCFs (ES2H) remained similar in ES2 cells (Figure 4, right two lanes). The volcano diagram demonstrated that the expression levels of 411 genes differed between the control and tumors treated with HCFs in SKOV3ip1 cells. HCFs upregulated 164 genes and downregulated 247 genes compared to those in the control tumor (Figure 5, left panel). In ES2 cells, 76 genes were significantly differentially expressed, and 19 genes were upregulated, and 57 genes were downregulated in HCFs (Figure 5, right panel).

**Figure 6:**
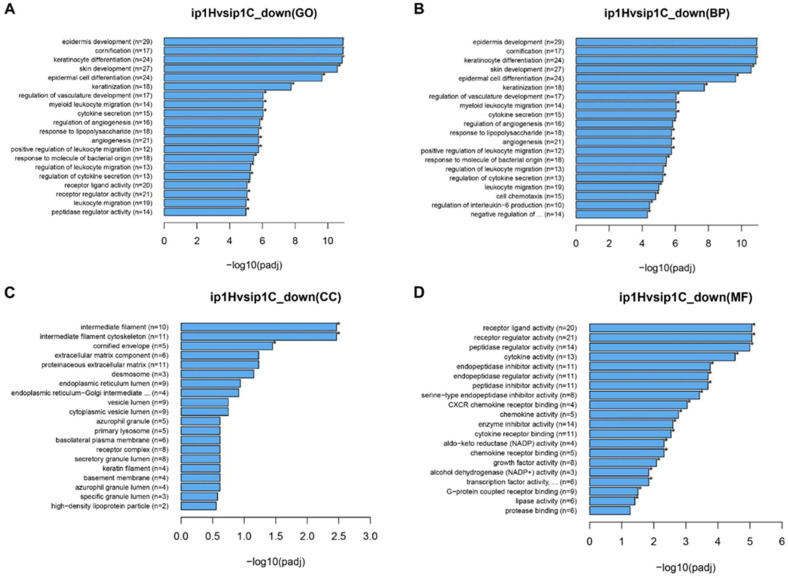
Top 20 enriched genes in the GO enrichment analysis in paired tumor treated with HCFs and control tumors not treated with films. Tumor tissues were obtained from the control (ip1C) and HCFs with 12–16 μm pores (ip1H) in SKOV3ip1 cells. (**A**) The top 20 ranked GO gene sets were downregulated in tumors treated with HCFs (ip1H) compared with that in the control (ip1C). (**B**) The top 20 ranked GO gene sets were downregulated in biological process (BP), (**C**) cellular comparison (CC), and (**D**) molecular function (MF). *, *p* < 0.05.

Next, we performed GSEA in SKOV3ip1 and ES2 cells to identify differences in molecular characteristics between the control and tumors treated with HCFs. GO gene sets were used in GSEA. In ES2 cells, only two pathways (GO0003707 and GO0005496) were significantly downregulated in tumors treated with HCFs (ES2H) compared with that in the control (ES2C). GO analysis was insufficient in ES2 cells to identify differences in molecular characteristics between the control and tumor treated with HCFs. In SKOV3ip1 cells, only two categories (GO0004620 and GO0016298) were significantly upregulated in tumors treated with HCFs (ip1H) compared to that in the control (ip1C). In contrast, the top 20 ranked GO gene sets that were downregulated in tumors treated with HCFs (ip1H) were significantly different than that in the control (ip1C) (Figure 6A). Significant enrichment of epidermis (GO0070268, GO0008544, GO0030216, GO0043588, GO0009913, and GO0031424)-, cytokine secretion (GO0050663, GO0050707, and GO0032675)-, angiogenesis (GO1901342, GO0045765, and GO0001525)-, leukocyte migration (GO0097529, GO0002687, GO0002685, GO0050900)-, and cell chemotaxis (GO0060326)-associated pathways was observed in tumors treated with HCFs (ip1H). The top 20 GO terms and clusters of genes downregulated by HCFs are listed in Table 1. GO includes three main branches: biological processes (BP), cellular comparison (CC), and molecular function (MF). Most GO terms in BP overlapped with the top 20 ranked GO gene sets (Figure 6B). Only three categories in CC were significant (Figure 6C), and most MF terms were significant in tumors treated with HCFs compared to the control (Figure 6D). The clusters of genes in MF were associated with cytokines, chemokines, G-protein coupled, and growth factor receptors (Table 2). Although one possible mechanism underlying tumor growth inhibition involves apoptosis induction by HCFs, no significant enrichment of apoptosis-associated pathways were observed in tumors treated with HCFs. Other possible mechanisms underlying cancer cell growth inhibition, including cell proliferation and focal adhesions, were described previously [19,22,23,24]. The included GO terms were significantly downregulated and are shown in Table 3. The significant categories related to cell proliferation and focal adhesions were 14 and 3, respectively.

**Figure 7:**
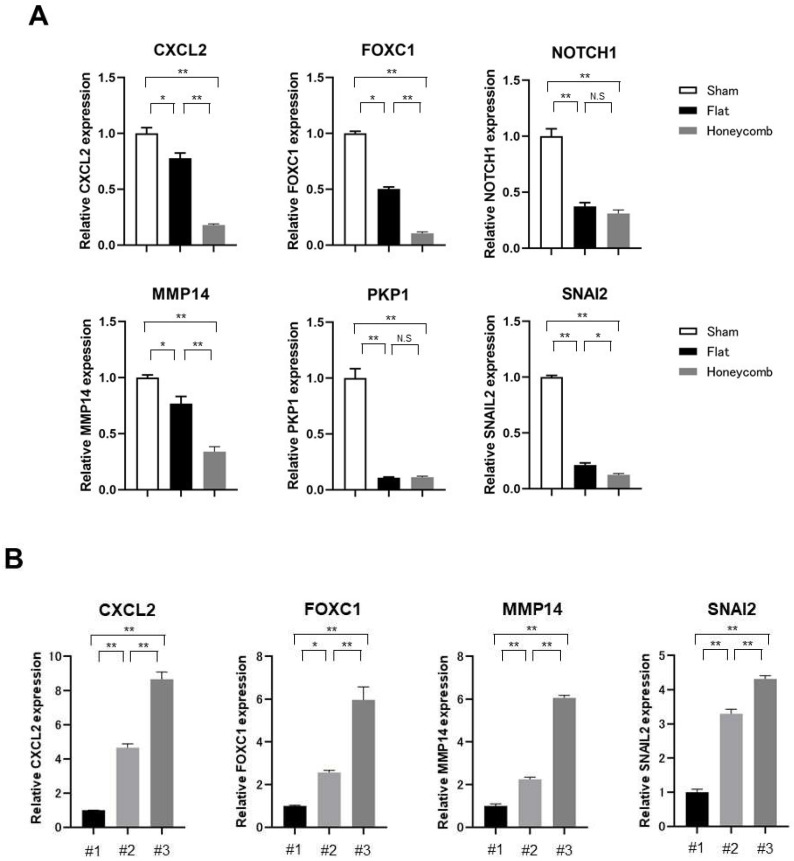
Expression of representative genes is associated with significant enrichments of GO pathways. (**A**) CXCL2, FOXC1, NOTCH1, PKP1, MMP14, and SNAI2 expressions were assessed using using qRT-PCR in control tumor not treated with films (Sham), tumors treated with flat films (Flat) and HCFs (Honenycomb). Gene expression levels were calculated from the ratio of the gene expression levels of tumor not treated with films (Sham) (set as 1) (n = 3). (**B**) CXCL2, FOXC1, MMP14, and SNAI2 expressions were assessed using using qRT-PCR inthe bottom (#1), middle (#2), and top (#3) of tumor sections. Gene expression levels were calculated from the ratio of the gene expression levels of tumor sections of right side (#1). Values are shown as the mean ± SE. Significant differences are indicated by asterisks. **, *p* < 0.01, *, *p* < 0.05. N.S; not significant.

Next, we selected representative genes associated with the significant enrichment of GO pathways and examined the expression levels of these genes in tumors treated with HCFs compared to that in the control, tumors treated with flat films and HCFs using qRT-PCR in SKOV3ip1 cells. CXCL2, FOXC1, and NOTCH1 involved in cell proliferation, migration, invasion, and angiogenesis [25,26,27]. PKP1, MMP14, and SNAI2 mediate focal adhesion, ECM, and epithelial–mesenchymal transition (EMT) [28,29,30]. CXCL2, FOXC1, NOTCH1, PKP1, MMP14, and SNAI2 expressions were significantly downregulated in tumors treated with flat and HCFs compared to those in the control (Figure 7A). Notably, CXCL2, FOXC1, MMP14, and SNAI2 were significantly downregulated in tumors treated with HCFs compared to flat films. These findings suggest that the films coming into contact with the tumor reduced those genes’ expression, and the genes dowregulated by the honeycomb structure were limited. We also exmained whether there is a gradient of gene expresion change that spreads across the tumor. The formed tumors treated with HCFs were divided into three sections, and the bottom, middle, and top of tumor sections were labelled numbers 1, 2, and 3, respectively. The gene expression levels of CXCL2, FOXC1, MMP14, and SNAI2 were compared in the three samples. The expression levels of those genes were significantly upregulated in an orderly manner in numbers 1, 2, and 3 (Figure 7B). Although the adhesive surface of HCFs was not evident from the preservation of tumors, the gradient of gene expression changes were observed for all genes downregulated by honeycomb structure.

## 4. Discussion

The results of the present study demonstrated that HCFs suppress tumor growth, and the appropriate pore size of HCFs to inhibit tumor growth is not related to the size of cancer cells. We also showed that HCFs transform cancer cells into rounded cells and maintain them in the pores. Based on our data, the mechanisms underlying tumor growth inhibition by HCFs might be identified as: (1) focal adhesion inhibition, (2) morphological changes induction, and (3) subsequent cell proliferation, migration, and differentiation suppression. The effect of HCFs on tumor growth may be associated with mechanosensors that sense mechanical information and converts it into electrical or chemical signals [31,32]. We also confirmed the downregulation of representative genes associated with significant enrichment of GO pathways, such as CXCL2, FOXC1, NOTCH1, PKP1, MMP14, and SNAI2. These genes are involved in cell proliferation, migration, focal adhesion, and EMT [25,26,27,28,29,30]. In these genes, CXCL2, FOXC1, MMP14, and SNAI2 were downregulated by the honeycomb structure. Collectively, our findings indicate that HCFs could be a novel device, inhibiting residual tumor growth, invasion, and metastasis after PDS. HCFs can reduce surgical invasiveness and improve the prognosis of patients with advanced ovarian cancer.

Although complete surgery with no residual disease results in better survival than suboptimal surgery with residual disease [3,4], the achievement of complete surgery is difficult due to widespread peritoneal dissemination in advanced ovarian cancer. To increase the complete surgery rate, neoadjuvant chemotherapy followed by interval debulking surgery (IDS) were administrated to patients with advanced ovarian cancer. In phase III clinical trials comparing PDS and IDS, the complete surgery rates were 12–47.6 and 39–77% for PDS and IDS, respectively [33,34,35,36]. In another study, the complete surgery rates were 60% and 24.6% in oncological referral centers and non-oncological referral centers, respectively [9]. In the present study, HCFs significantly inhibited tumor growth in an ovarian cancer mouse model. Therefore, applying HCFs to residual tumors may improve the prognosis of patients with advanced ovarian cancer who have undergone suboptimal surgery. HCFs may also be useful for patients treated in facilities where more aggressive surgery is not possible, and HCFs result in a reduction in surgical invasiveness. 

Recently, maintenance therapy incorporating inhibitors against poly(adenosine diphosphate (ADP)-ribose) polymerase (PARP) was administered to improve survival in patients with ovarian cancer [37,38,39]. Using maintenance therapy with a PARP inhibitor provide more favorable progression-free survival among women with high-grade serous carcinoma (HGSC) and a BRCA1/2 mutation and homologous-recombination deficiency (HRD). However, evidence of the efficacy of PARP inhibitors in other histological subtypes such as endometrioid, clear cell, and mucinous carcinoma, is lacking. Our study showed that HCFs inhibited tumor growth in SKOV3ip1, which is a serous carcinoma cell line, and ES-2, which is a clear cell carcinoma cell line. These results suggest that HCFs can be widely used in patients with ovarian cancer, regardless of the histological subtype. However, GO analysis of ES2 cells was insufficient to identify the common mechanism underlying tumor growth inhibition by HCFs in both cell lines. This is a limitation of this study. Further studies are needed to identify common changes in gene expression by HCFs, which can explain the effectiveness of HCFs in tumor growth suppression regardless of histological type.

In our study, the pore sizes that significantly inhibited tumor growth were different between SKOV3ip1 and ES-2 cells. There was no difference in the number of cells settled into the pores in HCFs of different pore sizes in the two cells. Thus, the inhibitory effects in HCFs of different pore sizes could not be explained by the size of cancer cells. The pore size on the surface of HCFs affects the morphology, proliferation, differentiation and function of various normal cells [14,15,16], and the pore size is an important factor in determining the effect of HCFs on the cells. In normal cells, HCFs enhanced cell viability, growth, function, and differentiation, whereas human cancer cells cultured on HCFs showed reduced cell growth and motility [19]. It remains unclear why HCFs have inconsistent effects on the growth of normal and cancer cells. HCFs have the common effect of focal adhesion and morphology, whereas they have the exact opposite effect on cell proliferation, in both cells. This could be attributed to differences in the mechanosensor signaling between normal and cancer cells. An in vitro study demonstrated that some cancer cells infiltrated the pores of HCFs and were trapped [19]. These results are similar to our finding that SKOV3ip1 cells cultured on HCFs with large pores appeared to be rounded and settled into the pores. Cells infiltrated the pore of HCFs were observed in ES2 cells by CLSM. There might be differences in the mechanosensor signaling between SKOV3ip1 and ES2 cells. 

In our study, HCFs were fabricated from polyurethane because it has excellent mechanical strength and elasticity. HCFs prepared from polyurethane showed no adverse effects such as skin symptoms and weight loss, and were stable throughout the study duration. Although HCFs prepared from polyurethane are biocompatible, they are non-degradable [40]. The ideal HCFs to control residual tumor after surgery should remain at the residual tumor region during the critical period of inhibiting tumor growth and be degradable in vivo after that period. HCFs fabricated from poly(lactic acid) (PLA) have better biodegradability than those from polyurethane [41,42], and thermoplastic polyurethanes with excellent biodegradability are reported to be developed [40]. Therefore, further studies are needed to identify the best chemical component of the polymer to control residual tumor after surgery. The inhibitory effect of HCFs on tumor growth is due to their porous structure, and similar results are likely to be obtained when HCFs are fabricated from other chemical compounds of the polymer.

Based on our GO analysis and qPCR results, HCFs decreased the expression of genes involved in focal adhesion, ECM, cell migration, proliferation, and EMT. However, simply applying flat films suppressed the expression of these genes and the genes downregulated by the honeycomb structure were limited. Moreover, there was a gradient of gene expression change that spreads across tumors. HCFs could cause downregulations in gene expression such as CXCL2, FOXC1, MMP14, and SNAI2 in the tumors contacted with the matrices. The upregulation of these genes might be observed in the middle and distant tumors from HCFs compared with the bottom surfaces of the formed tumors. Therefore, cell-to-cell signaling mechanisms may be important in inhibiting overall tumor growth. The reasons why HCFs suppressed tumor growth in ovarian cancer could be explained by the following possibilities. HCFs provide mechanical stress to ovarian cancer cells through locks in the pores, and then induce abnormal focal adhesion. Abnormal focal contacts may induce cell-shape deformation and have negative regulatory effects on cell migration and proliferation through the downregulation of chemokines and growth receptors. A previous study reported that HCFs do not increase apoptotic markers, such as caspase-3/7 and terminal deoxynucleotidyl transferase dUTP-biotin nick end labeling (TUNEL), but result in lower cyclin D1 expression and higher retinoblastoma protein expression [19]. In our study, no significant enrichment of apoptosis and cell-cycle-associated pathways was observed in tumors treated with HCFs. Therefore, HCFs may inhibit tumor growth by placing cancer cells in a dormant state. A limitation of our study is that the mechanism underlying tumor growth inhibition by HCFs in ES2 cells remains unclear. We could not show enough evidence to prove this. Another is the use of experimental methods of subcutaneous injection for ovarian cancer with widespread peritoneal dissemination. Further studies in an intraperitoneal administration are needed to advance the field.

## 5. Conclusions

We found that HCFs inhibited tumor growth in an ovarian cancer mouse model and that the appropriate pore size of HCFs to inhibit tumor growth was different according to the type of ovarian cancer cell line. Based on RNA sequencing data, the abnormal focal adhesion induced by honeycomb-like structures may alter cell morphology, subsequently inhibiting differentiation, proliferation and motility in ovarian cancer cells. Our data suggest that HCFs can be widely used for patients with ovarian cancer, regardless of their genomic status and histological subtypes. However, this is the first study to examine the effects of HCFs on tumor growth in using an in vivo ovarian cancer model. Further studies are needed to apply HCFs in the clinical stage. HCFs could be a novel device, inhibiting residual tumor growth after primary debulking surgery, and could reduce surgical invasiveness and improve the prognosis for patients with advanced ovarian cancer.

## Figures and Tables

**Table 1:** The top 20 ranked GO gene sets.

Category	ID	Description	*p* Value	padj	geneID	Count
BP	GO:0070268	cornification	4.04 × 10^−15^	1.18 × 10^−11^	PKP1/KRT23/PERP/CSTA/PI3/KRT17/SPINK5/DSC3/KRT7/TCHH/KLK13/KRT24/KRT27/KRT5/KRT16/KRT14/KRT6A	17
BP	GO:0008544	epidermis development	7.88 × 10^−15^	1.18 × 10^−11^	FOXC1/COL17A1/TP63/PKP1/PTHLH/FERMT1/KRT23/VDR/PERP/CSTA/PI3/EREG/KRT17/SPINK5/DSC3/KRT7/ZNF750/NOTCH1/TCHH/FOXQ1/KLK13/KRT24/KRT27/SFN/ALOX15B/KRT5/KRT16/KRT14/KRT6A	29
BP	GO:0030216	keratinocyte differentiation	1.31 × 10^−14^	1.31 × 10^−11^	FOXC1/TP63/PKP1/KRT23/VDR/PERP/CSTA/PI3/EREG/KRT17/SPINK5/DSC3/KRT7/NOTCH1/TCHH/KLK13/KRT24/KRT27/SFN/ALOX15B/KRT5/KRT16/KRT14/KRT6A	24
BP	GO:0043588	skin development	3.47 × 10^−14^	2.59 × 10^−11^	FOXC1/TP63/PKP1/FERMT1/KRT23/VDR/PERP/CSTA/PI3/EREG/KRT17/SPINK5/DSC3/KRT7/NOTCH1/TCHH/FOXQ1/KLK13/KRT24/KRT27/SFN/ALOX15B/KRT5/KRT16/KRT14/GJB3/KRT6A	27
BP	GO:0009913	epidermal cell differentiation	3.77 × 10^−13^	2.25 × 10^−10^	FOXC1/TP63/PKP1/KRT23/VDR/PERP/CSTA/PI3/EREG/KRT17/SPINK5/DSC3/KRT7/NOTCH1/TCHH/KLK13/KRT24/KRT27/SFN/ALOX15B/KRT5/KRT16/KRT14/KRT6A	24
BP	GO:0031424	keratinization	3.52 × 10^−11^	1.75 × 10^−8^	PKP1/KRT23/PERP/CSTA/PI3/KRT17/SPINK5/DSC3/KRT7/TCHH/KLK13/KRT24/KRT27/SFN/KRT5/KRT16/KRT14/KRT6A	18
BP	GO:0050663	cytokine secretion	2.41 × 10^−9^	9.01 × 10^−7^	FERMT1/IL1A/GBP1/CD274/IL1B/CHI3L1/IL6/IL33/CASP1/NOTCH1/ZC3H12A/SAA1/ALOX15B/IL1RAP/CARD16	15
BP	GO:0097529	myeloid leukocyte migration	2.66 × 10^−9^	9.01 × 10^−7^	CXCL2/CHGA/SERPINE1/IL1A/CCL20/PGF/IL1B/IL6/MMP14/CXCL3/CXCL1/CXCL8/SAA1/CSF1	14
BP	GO:1901342	regulation of vasculature development	2.72 × 10^−9^	9.01 × 10^−7^	FOXC1/NGFR/SERPINE1/IL1A/PGF/IL1B/C3/ID1/SERPINF1/CHI3L1/SPINK5/IL6/NOTCH1/ZC3H12A/HEY1/CXCL8/MMRN2	17
BP	GO:0045765	regulation of angiogenesis	4.79 × 10^−9^	1.43 × 10^−6^	FOXC1/NGFR/SERPINE1/IL1A/PGF/IL1B/C3/ID1/SERPINF1/CHI3L1/SPINK5/IL6/NOTCH1/ZC3H12A/CXCL8/MMRN2	16
BP	GO:0002687	positive regulation of leukocyte migration	6.28 × 10^−9^	1.70 × 10^−6^	CXCL2/BDKRB1/SERPINE1/IL1A/CCL20/PGF/IL6/MMP14/CXCL3/CXCL1/CXCL8/CSF1	12
BP	GO:0001525	angiogenesis	7.23 × 10^−9^	1.70 × 10^−6^	FOXC1/NGFR/CALCRL/SERPINE1/IL1A/PGF/EREG/IL1B/C3/ID1/SERPINF1/CHI3L1/SPINK5/IL6/NOTCH1/MMP14/ZC3H12A/HEY1/CXCL8/MMRN2/NOTCH4	21
BP	GO:0032496	response to lipopolysaccharide	7.41 × 10^−9^	1.70 × 10^−6^	CD6/NGFR/CXCL2/BDKRB1/SERPINE1/CCL20/IL1B/IRAK2/IL6/CASP1/NOTCH1/CXCL3/CXCL1/ZC3H12A/CXCL8/THBD/CD55/CARD16	18
BP	GO:0002237	response to molecule of bacterial origin	1.61 × 10^−8^	3.44 × 10^−6^	CD6/NGFR/CXCL2/BDKRB1/SERPINE1/CCL20/IL1B/IRAK2/IL6/CASP1/NOTCH1/CXCL3/CXCL1/ZC3H12A/CXCL8/THBD/CD55/CARD16	18
BP	GO:0002685	regulation of leukocyte migration	2.63 × 10^−8^	5.24 × 10^−6^	CXCL2/BDKRB1/SERPINE1/IL1A/CCL20/PGF/IL6/IL33/MMP14/CXCL3/CXCL1/CXCL8/CSF1	13
BP	GO:0050707	regulation of cytokine secretion	3.04 × 10^−8^	5.67 × 10^−6^	FERMT1/IL1A/GBP1/CD274/IL1B/IL6/IL33/CASP1/ZC3H12A/SAA1/ALOX15B/IL1RAP/CARD16	13
BP	GO:0050900	leukocyte migration	5.64 × 10^−8^	9.90 × 10^−6^	CXCL2/CHGA/BDKRB1/SERPINE1/IL1A/CCL20/PGF/IL1B/IL6/IL33/SLC7A7/MMP14/CXCL3/CXCL1/INPP5D/CXCL8/SAA1/THBD/CSF1	19
BP	GO:0060326	cell chemotaxis	9.33 × 10^−8^	1.55 × 10^−5^	CXCL2/CHGA/SERPINE1/CCL20/PGF/IL1B/SAA2/IL6/NOTCH1/CXCL3/CXCL1/CXCL8/SAA1/CSF1/PLXNB3	15
BP	GO:0032675	regulation of interleukin-6 production	2.27 × 10^−7^	3.57 × 10^−5^	BPI/IL1A/EREG/IL1B/IL6/IL33/ZC3H12A/INPP5D/ADORA2B/IL1RAP	10
BP	GO:0010951	negative regulation of endopeptidase activity	3.25 × 10^−7^	4.86 × 10^−5^	NGFR/TFPI2/SERPINE1/CSTA/PI3/C3/SERPINF1/SPINK5/IL6/SFN/SERPINA3/SERPINA1/CARD16/SERPINB5	14

**Table 2:** The GO gene sets in molecular function involving receptors.

Category	ID	Description	*p* Value	padj	geneID	Count
MF	GO:0005126	cytokine receptor binding	1.07 × 10^−5^	0.000916	CXCL2/IL1A/CCL20/TNFSF4/PGF/IL1B/IL6/INHBB/CXCL3/CXCL1/IL13/CXCL8/CSF1/LTA/ITGB3	15
MF	GO:0045236	CXCR chemokine receptor binding	8.79 × 10^−5^	0.004524	CXCL2/CXCL3/CXCL1/CXCL8	4
MF	GO:0001664	G-protein coupled receptor binding	0.0005354	0.017232	GNA15/CXCL2/RNF43/CCL20/C3/SHANK1/ADORA1/CXCL3/CXCL1/BDKRB2/CXCL8/SAA1	12
MF	GO:0042379	chemokine receptor binding	0.0009311	0.023976	CXCL2/CCL20/CXCL3/CXCL1/CXCL8	5
MF	GO:0070851	growth factor receptor binding	0.0010385	0.025467	AREG/IL1A/PGF/EREG/IL1B/FLRT1/IL6/ITGB3	8
MF	GO:0030276	clathrin binding	0.002324	0.047874	LRP1/TRPC6/SYT8/DOC2A/DNER	5

**Table 3:** The GO gene sets involving cell proliferation and focal adhesion.

Category	ID	Description	*p* Value	padj	geneID	Count
BP	GO:0043616	keratinocyte proliferation	2.23 × 10^−5^	0.0015763	SNAI2/TP63/FERMT1/VDR/EREG/SFN	6
BP	GO:0050678	regulation of epithelial cell proliferation	5.68 × 10^−5^	0.0028436	SNAI2/NGFR/TP63/VDR/PGF/EREG/ID1/DLL4/SERPINF1/IL6/NOTCH1/SFN/PLXNB3/SERPINB5/ITGB3	15
BP	GO:0014009	glial cell proliferation	0.0002361	0.007432	AREG/IL33/NOTCH1/ASCL2/LTA	5
BP	GO:0050673	epithelial cell proliferation	2.17 × 10^−6^	0.0002763	SNAI2/NGFR/TP63/FERMT1/AREG/VDR/PGF/EREG/ID1/DLL4/SERPINF1/IL6/LGR5/NOTCH1/MMP14/SFN/PLXNB3/SERPINB5/ITGB3	19
BP	GO:0032946	positive regulation of mononuclear cell proliferation	0.0002448	0.0075068	CD6/TNFSF4/CD274/IL1B/IL6/CD1D/IL13/CSF1/CD55	9
BP	GO:0070665	positive regulation of leukocyte proliferation	0.000323	0.0091505	CD6/TNFSF4/CD274/IL1B/IL6/CD1D/IL13/CSF1/CD55	9
BP	GO:2000647	negative regulation of stem cell proliferation	0.0005807	0.0137109	SNAI2/FERMT1/KCTD11	3
BP	GO:0010839	negative regulation of keratinocyte proliferation	0.0007653	0.0167911	SNAI2/VDR/SFN	3
BP	GO:0010837	regulation of keratinocyte proliferation	0.000794	0.017078	SNAI2/TP63/VDR/SFN	4
BP	GO:0042102	positive regulation of T cell proliferation	0.0008037	0.017078	CD6/TNFSF4/CD274/IL1B/IL6/CD1D/CD55	7
BP	GO:0050671	positive regulation of lymphocyte proliferation	0.0011127	0.0213732	CD6/TNFSF4/CD274/IL1B/IL6/CD1D/IL13/CD55	8
BP	GO:0032944	regulation of mononuclear cell proliferation	0.0014005	0.0239278	CD6/TNFSF4/CD274/IL1B/IL6/CD1D/INPP5D/IL13/CSF1/CD55	10
BP	GO:0070663	regulation of leukocyte proliferation	0.0018681	0.028611	CD6/TNFSF4/CD274/IL1B/IL6/CD1D/INPP5D/IL13/CSF1/CD55	10
BP	GO:0050680	negative regulation of epithelial cell proliferation	0.0021854	0.0323065	SNAI2/NGFR/VDR/EREG/DLL4/SERPINF1/SFN	7
BP	GO:0010812	negative regulation of cell-substrate adhesion	0.0014459	0.0244574	SERPINE1/GBP1/NOTCH1/MMP14/MELTF	5
BP	GO:0030198	extracellular matrix organization	0.0021733	0.0322666	TNC/ELN/FOXC1/FERMT1/SERPINE1/LRP1/SPINK5/NOTCH1/MMP14/MELTF/TMPRSS6/SERPINB5/ITGB3	13
BP	GO:0045109	intermediate filament organization	0.0036129	0.0448321	PKP1/KRT17/KRT14	3
BP	GO:0045104	intermediate filament cytoskeleton organization	0.0004401	0.0116361	PKP1/KRT17/KRT16/KRT14/KRT6A	5

## Data Availability

The datasets used and/or analyzed during the current study are available from the corresponding author on reasonable request.

## Supplementary Materials

The following supporting information can be downloaded at: https://www.mdpi.com/article/10.3390/cancers15010237/s1, Table S1: Primer sequences for qPCR.
