# Supplementary figures and images for: Honeycomb-like Structured Film, a Novel Therapeutic Device, Suppresses Tumor Growth in an In Vivo Ovarian Cancer Model
Source: Cancers (Basel). 2022 Dec 30;15(1):237. doi: 10.3390/cancers15010237 (PMC9818543; doi:10.3390/cancers15010237)

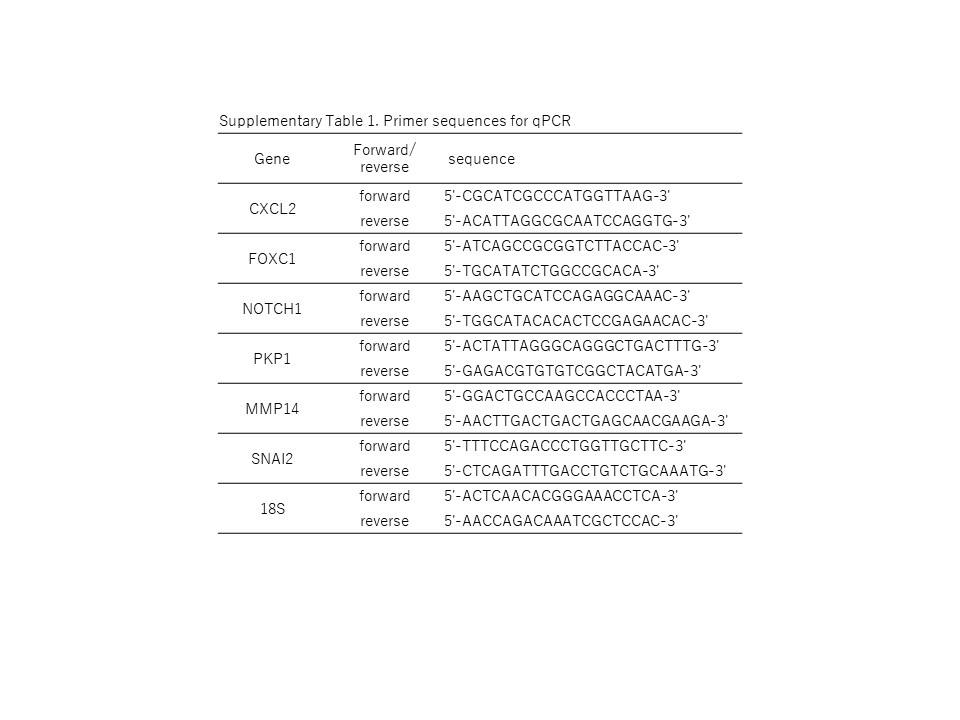

Supplement: Supplementary file 1 [file cancers-15-00237-s001.zip › cancers-1949800-supplementary.jpg]
